# Supplementary material for: Ionic Liquid-Polypyrrole-Gold Composites as Enhanced Enzyme Immobilization Platforms for Hydrogen Peroxide Sensing
Source: Sensors (Basel). 2019 Feb 3;19(3):640. doi: 10.3390/s19030640 (PMC6387225; doi:10.3390/s19030640)
Supplement: Supplementary file 1 [file sensors-19-00640-s001.pdf]

Ionic Liquid-Polypyrrole-Gold Composites as Enhanced Enzyme Immobilization Platforms for Hydrogen Peroxide Sensing

Meng Li [1], Jing Wu [1], Haiping Su [1], Yan Tu [1], Yazhuo Shang [1,*], Yifan He [2,*], Honglai Liu [1]

[1] Key Laboratory for Advanced Materials, School of Chemistry and Molecular Engineering, East China University of Science and Technology, Shanghai 200237, China; shangyazhuo@ecust.edu.cn

[2] Department of Biotechnology, School of Sciences, Beijing Technology and Business University, Beijing 100048, China; heyifan@btbu.edu.cn

* Correspondence: shangyazhuo@ecust.edu.cn; Tel.: +86-21-6425-2767 (Y.Z.S.)

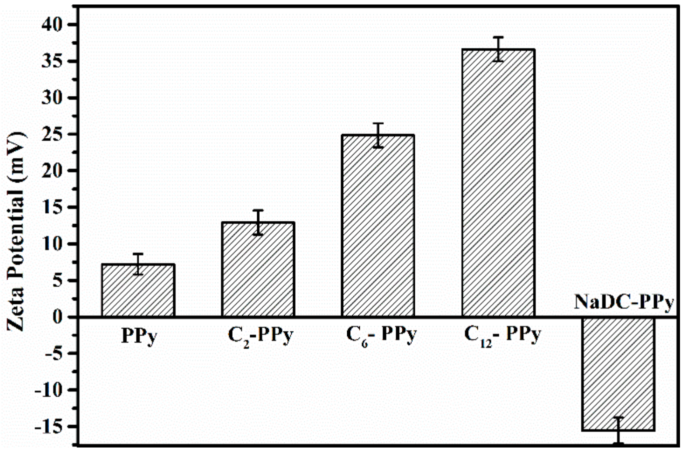

Figure S1. Zeta potential of different samples prepared in water, [C_2min]Br (1 M), [C_6min]Br (1 M), [C_{12}min]Br (1M), NaDC (0.5 M) solutions respectively.

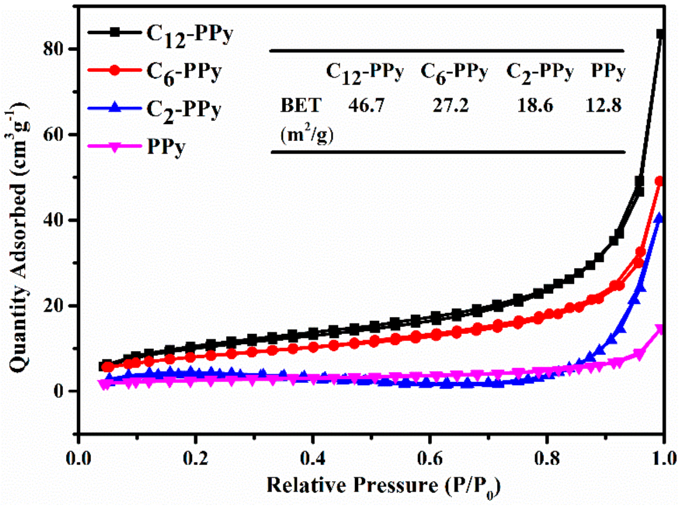

Figure S2. Nitrogen adsorption-desorption isotherms and BET surface area (inset) of C_{12}-PPy, C_6-PPy, C_2-PPy and PPy.

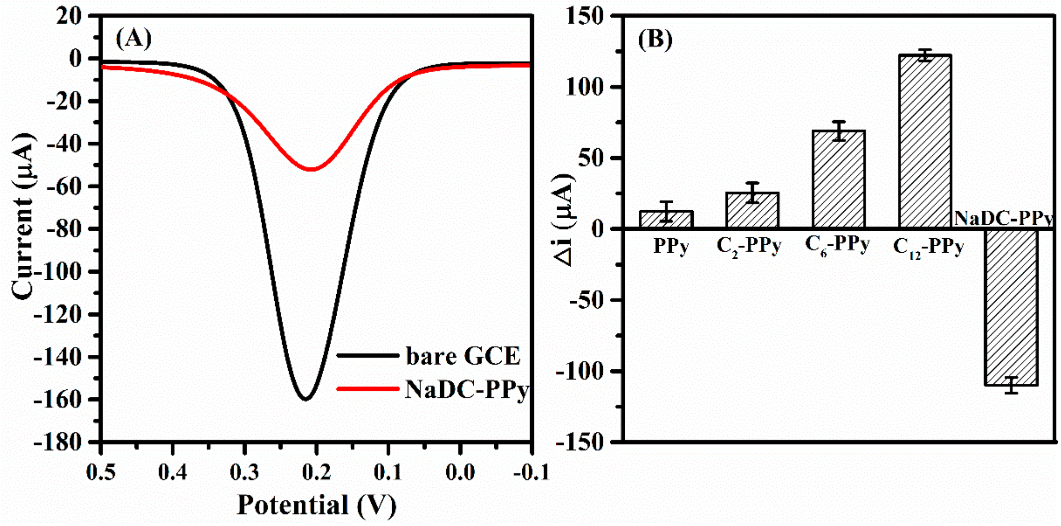

Figure S3. (**A**) DPV response of bare GCE and NaDC-PPy/GCE in 0.01 M PBS containing 5 mM $Fe[(CN)_6]^{3-/4-}$ and 0.1 M KCl (pH 7.0). (**B**) Comparison of current response variation of GCE modified by different material.

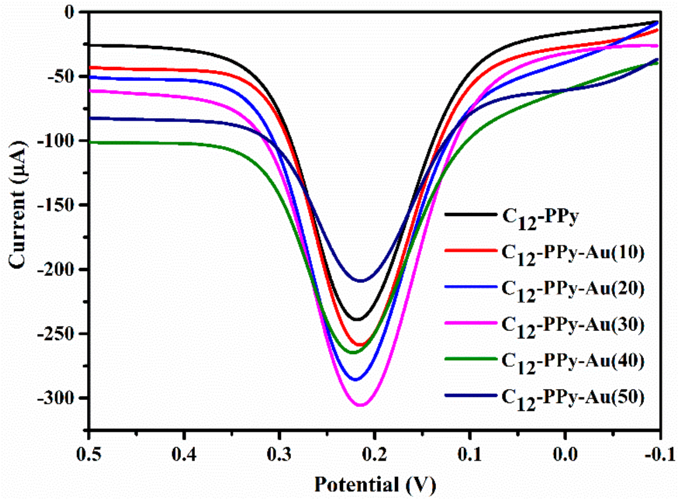

Figure S4. DPV response of C_{12}-PPy-Au/GCE obtained by scanning C_{12}-PPy/GCE for 10, 20, 30, 40, and 50 segments in 1 mM $HAuCl_4$ solution at a scan rate of 50 mVs^{-1} respectively.
